# Evaluation of the remineralizing capacity of silver diamine fluoride on demineralized dentin under pH-cycling conditions

**DOI:** 10.1590/1678-7757-2022-0306

**Published:** 2023-03-27

**Authors:** Carolina Cecilia CIFUENTES-JIMÉNEZ, María Victoria BOLAÑOS-CARMONA, Tattiana ENRICH-ESSVEIN, Santiago GONZÁLEZ-LÓPEZ, Pedro ÁLVAREZ-LLORET

**Affiliations:** 1 Universidad de Granada Facultad de Odontología Departamento de Estomatología Granada España Universidad de Granada, Facultad de Odontología, Departamento de Estomatología, Granada, España.; 2 Universidad de Granada Facultad de Odontología Departamento de Pediatría Granada España Universidad de Granada, Facultad de Odontología, Departamento de Pediatría, Granada, España.; 3 Universidad de Oviedo Facultad de Geología Oviedo España Universidad de Oviedo, Facultad de Geología, Oviedo, España.; 4 Universidad de Granada Facultad de Ciencias Departamento de Mineralogía y Petrología Granada España Universidad de Granada, Facultad de Ciencias, Departamento de Mineralogía y Petrología, Granada, España.

**Keywords:** Dentin, Sodium fluoride, Biomineralization, Dental caries, Silver compounds, Silver diamine, Crystallization

## Abstract

**Objective:**

(1) to determine the effects of the silver diamine fluoride (SDF) and sodium fluoride (NaF) in demineralized dentin exposed to an acid challenge by pH-cycling, (2) to evaluate the remineralizing capacity of SDF/NaF products based on the physicochemical and mechanical properties of the treated dentin surfaces.

**Methodology:**

In total, 57 human molars were evaluated in different stages of the experimental period: sound dentin – negative control (Stage 1), demineralized dentin – positive control (Stage 2), and dentin treated with SDF/NaF products + pH-c (Stage 3). Several commercial products were used for the SDF treatment: Saforide, RivaStar, and Cariestop. The mineral composition and crystalline and morphological characteristics of the dentin samples from each experimental stage were evaluated by infrared spectroscopy (ATR-FTIR), X-ray diffraction, and electron microscopy (SEM-EDX) analytical techniques. Moreover, the mechanical response of the samples was analyzed by means of the three-point bending test. Statistics were estimated for ATR-FTIR variables by Wilcoxon test, while the mechanical data analyses were performed using Kruskal-Wallis and Mann Whitney U tests.

**Results:**

Regarding the chemical composition, we observed a higher mineral/organic content in the SDF/NaF treated dentin + pH-c groups (Stage 3) than in the positive control groups (Saforide p=0.03; Cariestop p=0.008; RivaStar p=0.013; NaF p=0.04). The XRD results showed that the crystallite size of hydroxyapatite increased in the SDF/NaF treated dentin + pH-c groups (between +63% in RivaStar to +108% in Saforide), regarding the positive control. SEM images showed that after application of the SDF/NaF products a crystalline precipitate formed on the dentin surface and partially filled the dentin tubules. The flexural strength (MPa) values were higher in the dentin treated with SDF/NaF + pH-c (Stage 3) compared to the positive control groups (Saforide p=0.002; Cariestop p=0.04; RivaStar p=0.04; NaF p=0.02).

**Conclusions:**

The application of SDF/NaF affected the physicochemical and mechanical properties of demineralized dentin. According to the results, the use of SFD/NaF had a remineralizing effect on the dentin surface even under acid challenge.

## Introduction

Dental caries is a major health problem in most countries, where the disease affects 60 to 90% of children and most adults.^1^ Although the prevalence of dental caries decreased over the past decade, it is still one of the most common diseases worldwide.^2^ In fact, dental caries majorly impacts the global clinical and economic burden, thus, an improved approach for its prevention and therapy is essential.^3^ Dental caries is a multifactorial, dynamic, and chronic disease characterized by demineralization and cavitation of susceptible dental hard tissues.^4^ The process involved in the caries development is biofilm mediated and sugar driven, resulting in the phasic demineralization and remineralization of tooth structures.^5^ At the inorganic level, the demineralization process during dental caries starts with the acidic dissolution of hydroxyapatite crystals, leading to the formation of cavities.

Dental caries in enamel and dentin can be remineralized and arrested at an early stage of the disease by using chemical products at clinical level. The minimally invasive approach to caries treatment should respect dental tissues and traumatize less the patients, avoiding surgery as long as possible and focusing on the preservation of natural tooth structure.^5 , 6^ Preventive and non-restorative treatments, such as the use of fluoride agents, are widely employed in the treatment of dental caries.^7 , 8^

For the application of fluorides, established protocols must be followed, with each fluoride in its own recommended concentration, frequency of use, and dosage schedule.^8^ Moreover, the use of fluoride products, such as toothpastes and mouthwashes, is becoming a common complement in oral care. Currently, the use of oral rinses containing 0.2% NaF may prevent caries.^9 , 10^ On the other hand, silver diamine fluoride (SDF) is a colorless, odorless, and alkaline topical biomaterial.^7^ The commercial market has different concentrations of SDF ( *i.e.,* 12%, 30%, and 38%) available.^8^ However, the most common SDF concentration is 38%, which contains a high concentration of fluoride (equivalent to a relative concentration of 5%; 44.800 ppm), silver ions (25%; 253.900 ppm), 8% ammonia, and 62% water.^11 , 12^ Since the effectiveness of the SDF with concentration of 38%, the product is used for management of dentin hypersensitivity, prevention of caries, pits, and fissures in adults and children, and arrest of root caries.^13^ Moreover, previous studies showed that SDF can prevent and arrest the formation of new caries lesions in young children as well as in older adults.^14 , 15^ Systematic reviews also concluded that SDF treatment achieves a 70 to 85% success rate in arresting pediatric caries.^16^ The uncomplicated SDF application protocol has minimal potential risk and is painless, allowing to treat apprehensive young children, disadvantaged population, and elderly.^7^

The mechanism by which SDF prevents and remineralizes dental caries is only partially understood.^17^ The preferred assumption is that the fluoride ions in the SDF help to remineralize the carious dentin, while the silver ions provide antibacterial activity and reduce collagen degradation by inhibiting dentin collagenases.^18^ Additionally, the high concentration of fluoride ions reacts with the dentin to form calcium fluoride, as silver reacts with chlorine or phosphate compounds of the dentin, leading to the formation of silver salts.^19^ This reaction was previously described in the immediate effects of SDF on demineralized dentin, resulting in the precipitation of different crystalline phases and increased mineralization on the dentin surface after the products application.^20^ Moreover, the use of SDF inhibits the proteolytic activity of the matrix metalloproteinases (MMPs) and prevents dentin collagen degradation from bacterial collagenase action.^13 , 17 , 21^ Although previous *in vitro* studies demonstrated the properties of SDF compounds in the treatment of caries, the effects of these fluoride products on demineralized dentin under acid challenge ( *i.e.* , pH-c process) were scarcely analyzed.^22 , 23^

The pH-cycling (pH-c) procedure, standardized by Marquezan, et al.^24^ (2009), is one of the best methods to simulate affected dentin caries layers. The procedure is easy to reproduce and cost-effective, showing high experimental control and sensitivity to variable treatment behavior.^25 , 26^ In this way, pH-c is appropriate to evaluate the demineralization effects on *in vitro* caries simulation and the response of dental materials ( *e.g.,* SDF products) for caries treatment in clinical practice. Therefore, the present study aimed (1) to determine the effects of the SDF/NaF in demineralized dentin exposed to an acid challenge by pH-c and, (2) to evaluate the remineralizing capacity of these products based on the physicochemical and mechanical properties of the treated dentin surfaces. For this purpose, we employed several analytical techniques to characterize the morphological, compositional, and crystalline properties of the dentin mineral surfaces at different experimental stages. Furthermore, we also determined the mechanical strength of the treated dentin to evaluate its possible clinical performance. The hypotheses were: (1) the use of SDF/NaF would not affect the physicochemical properties of the demineralized dentin after the pH-c and (2) the use of SDF/NaF would not affect the morphological structural of the demineralized dentin after the pH-c.

## Methodology

### Specimen preparation and experimental design

The study was approved by the local Ethics Committee on Human and Animal Research (Reference number #1020-2020). In total, 57 human permanent molar teeth (non-carious or morphological defects) were stored in 0.1% thymol solution at 4°C before preparation.

Tooth roots were separated from the crowns 2 mm below the cement-enamel junction using an Isomet 11/1180 low-speed saw (Buehler, Lake Bluff, IL, USA) with a 456CA diamond disk (Struers, Copenhagen, Denmark). Teeth slices (9 mm diameter x 1 mm thickness) were obtained by transversal sections of the mid-coronal for each tooth. Subsequently, four dentin beams (6x1x1 mm^3^) were obtained from the teeth slices, resulting in 228 beam-shaped specimens. The enamel was removed to result in a dentin beam. The samples were rinsed and cleaned by ultrasonication for 30 min to remove the remaining debris. Then, the specimens were carefully examined under an optical microscope to confirm the absence of microcracks and dental imperfections for further experiments. Finally, the samples corresponding to the negative control (Stage 1) were stored at room temperature and 90% relative humidity.

The carious dentin lesions were created using a pH-c procedure modified by Marquezan, et al.^24^(2009). All the specimens were immersed, for 8 hours, in 1 ml of a demineralizing solution containing 2.2 mM CaCl_2_, 2.2 mM NaH_2_PO_4_, and 50 mM acetic acid adjusted to a pH=4.8. Thereafter, samples were introduced, for 16 hours, in 1 ml of a remineralizing solution containing 1.5 mM CaCl_2_, 0.9 mM NaH_2_PO_4_, and 0.15 M KCl adjusted to a pH=7.0. Chemical reagents for solution preparation were supplied by Sigma-Aldrich, Saint Louis, MO, USA (≥99.0% purity). Each specimen was cycled for 14 days and the solutions were renewed daily. The pH-c was performed at room temperature without agitation.

**Figure 1:**
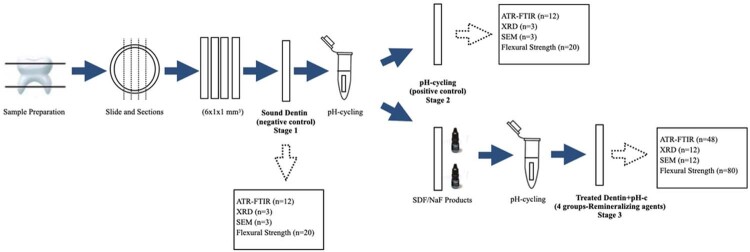
Schematic diagram of the experimental design

Subsequently, the demineralized dentin specimens were randomly divided into four experimental groups, according to the cariostatic remineralization products applied (commercial SDF): Saforide, Cariestop, RivaStar, and NaF treatments. Table 1 shows the product characteristics and application procedures. After the treatment with the different products, the samples were resubjected to pH-c for 14 days to evaluate the remineralizing effect of the products under an acid challenge ( *i.e., in vitro* caries process). In this study, the samples were analyzed during the different stages of the experimental period: sound dentin – negative control (Stage 1), demineralized dentin – positive control (Stage 2), and SDF/NaF treated dentin + pH-c (Stage 3), using several analytical techniques. Figure 1 summarizes the experimental method and design, showing the techniques employed for the compositional, microstructural, morphological, and mechanical characterization of dentin samples for each stage.

**Table 1:** Materials, composition, pH values, and application procedures of the silver diamine fluoride (SDF) and sodium fluoride (NaF) products

Material	Composition	pH	Application
Saforide 38% (Tokyo Seiyaku Kasei; Osaka, Japan)	Ammonium hydroxide, silver nitrate, hydrofluoric acid, water	13	Apply with a brush for 3 min. Rinse
Cariestop 30% (Biodinâmica Química e Farmacêutica; Ibipora, Brazil)	Ammonium hydroxide, silver nitrate, hydrofluoric acid, deionized water	12	Apply with a brush for 3 min. Rinse
RivaStar 31.3% (SDI; Bayswater, Victoria, Australia)	Capsule 1: Silver fluoride, ammonia solution Capsule 2: Potassium iodide	12.6	Capsule 1: Apply the solution with a brush to the treatment site only. Capsule 2: Apply a generous amount of the solution to the treatment site until the solution turns clear for a minute
Sodium Fluoride 0.2% (Farmacia Santamaría, Granada, Spain)	Sodium fluoride and deionized water	9	Apply with a brush for 3 min. Rinse

### Attenuated Total Reflectance-Fourier Transform Infrared (ATR-FTIR) spectroscopy

In total, 96 samples (n=24 spectra per group) were employed for the chemical characterization of dentin samples during the different stages of the experimental periods. Dentin samples were analyzed with a JASCO 6200 (Jasco, Easton, MD, USA) FTIR spectrometer equipped with a diamond-tipped ATR Pro ONE accessory. The spectra were recorded in absorption mode at a resolution of 2 cm^- 1^ over 124 scan accumulations with a spectral range from 600 to 4000 cm^- 1^. The absorption bands in the ATR-FTIR were associated to different molecular groups related to the chemical composition at molecular level of the dentin mineral ( *v*_1,_ *v*_3 _PO_3_^- 4^: phosphate band and amide I, II, III – organic matrix components). The overlapping peaks were resolved and their integrated areas were measured using a curve fitting software (Peakfit v4.12, Systat Software, San Jose, CA, USA). The second derivative method was used to resolve the peak estimations within each spectrum region. The degree of smoothing was adjusted to 10% (Savitzky-Golay algorithm) and a mixed Gaussian-Lorentzian function was used to adapt the profile of the peaks. The following parameters were calculated to describe dentin compositional properties: (1) the relative amount of mineral to the organic matrix (PO_4_/amide I) as the ratio of the main phosphate (PO_4_ stretch: 1030 cm^- 1^) to the amide I area ratio (type I collagen: 1640 cm^- 1^); (2) Crystallinity Index (CI) is the ratio between phosphate sub-bands areas at 1030 cm^- 1^ (high crystalline apatite phosphates) to 1020 cm^- 1^ (poorly crystalline apatite phosphates).^27^

### X-ray Diffraction (XRD) analyses

A convenience sample of three dentin beams was randomly selected and two-dimensional X-ray diffraction (2D-XRD) patterns were obtained per beam by using an X-ray diffractometer (Bruker D8 DISCOVER, Billerica, MA, USA), equipped with an area detector (DECTRIS PILATUS 3 100K-A). For the diffraction experiments, the working conditions were: Cu Kα (λ=1.5418 Å) radiation at 50 kV and 30 mA, with a pinhole collimator of 0.5 mm in diameter. The analyzed spots in the dentin surfaces were determined by an optical microscope equipped with a laser reference. Diffraction patterns were obtained from three different locations on the dentin surface. The 2D-XRD patterns were registered at 2Theta scanning angle range from 20° to 60°, considering 19 steps and 40 s/step. The intensities concentrated in arcs within the Debye diffraction rings (corresponding to specific d-spacing/diffraction lines) were integrated to obtain a unidimensional scan ( *i.e.,* 2Theta pattern).

The crystallite size for hydroxyapatite crystals of dentin mineral was determined by measuring the full width at half maximum (FWHM) of the diffraction peaks (002 reflection) displayed in 2Theta pattern. For this purpose, the Debye-Scherrer equation was used to estimate crystallite size: d=Kλ/β cos θ; where d is the mean size of the crystallites (expressed in nm), λ is the wavelength of the X-ray source, K is the Scherrer’s constant (K=0.89), and β is the FWHM of the line broadening for specific diffraction reflections. The determination of crystallite size using XRD techniques corresponds to the average measurement of volume of the crystalline domains in the specific *c-axes* direction.

### Scanning Electron Microscopy (SEM) analyses

To analyze the morphology and structure of the dentin under the different experimental stages, a convenience sample of three dentin specimens were randomly selected. All samples were fixed in glutaraldehyde solution after they were rinsed with PBS. Then, specimens were dehydrated in ascending ethanol series (50%, 70%, 90%, and 96%). Finally, samples were assembled on aluminum stubs and gold coated (approx. 20 nm thickness layer) using ion sputtering equipment. The sample surfaces were examined using a scanning electron microscopy (JSM-6610LV, JEOL, Japan) with an accelerating voltage of 20 kV and 10 mA and a magnification of 10.000–30.000x. Energy dispersive X-ray (EDX) spectroscopy was also obtained (Quanta 400, FEI, Hillsboro, OR, USA) to determine the elemental composition (Ca, P, Ag, I, C, O, and Na) on the dentin specimens, and three points per sample were measured.

### Flexural strength – mechanical test

The flexural properties of 120 beams (n=20) were measured using a universal testing machine (model 3345, Instron, Canton, MA, USA) by a three-point bending test on a two-point support with a 4.0 mm span length, employing a 500 N load cell at a crosshead speed of 0.5 mm/min until the sample fracture.

### Statistical analyses

Descriptive statistics were calculated for ATR-FTIR data and three-point bending test. After checking the normality of data distribution (Kolmogorov-Smirnov’s or Shapiro-Wilk’s tests, depending on the sample size), non-parametric tests allowed comparisons between groups or pairs. The comparisons between the ATR-FTIR data groups of the different experimental stages were performed by Wilcoxon test, while the mechanical data were carried on using Kruskal–Wallis and Mann Whitney U tests. A significance level of p<0.05 was established. Results were processed with SPSS statistical software (version 22, IBM0, Armonk, NY, USA). The sample size estimation and power analysis were performed using G*Power software (ver. 3.1.9.7; Heinrich-Heine-Universität Düsseldorf, Düsseldorf, Germany). The input parameters were: one-tailed test, 0.5 effect size d (medium), 0.05 α error level, and 0.6 power (1-β error). The results suggested a minimum sample size of 17 specimens for between-group comparisons by ATR-FTIR and mechanical testing.

## Results

### ATR-FTIR spectroscopic analysis

**Figure 2:**
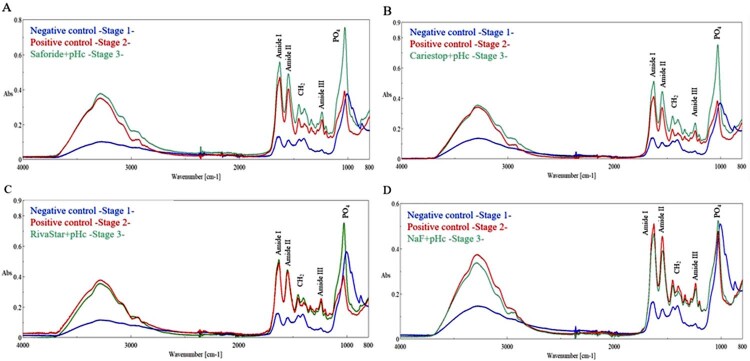
Representative ATR-FTIR spectra of negative control (Stage1), positive control (Stage2), and using remineralizing products (Stage3): (A) Saforide, (B) Cariestop, (C) RivaStar, and (D) NaF

Figure 2 shows a comparison between the ATR-FTIR spectra of the negative and positive controls and the treated SDF/NaF remineralizing groups. In all groups, we observed a loss of the mineral component ( *v*_1,_ *v*_3 _PO _3_^-4^; phosphate band) after the pH-c process (positive control and treated dentin groups). Furthermore, the positive control and SDF treated groups ( *i.e.,* Saforide, Cariestop, and RivaStar) showed a higher relative intensity in the absorption peaks corresponding to the organic components. Moreover, we observed a decreased intensity of amide I (1640 cm^-1^) and amide II (1550 cm^-1^), as well as the broadening and decreased intensity at 1400 cm^-1^ of amide III (1240 cm^-1^), in the SDF treated groups regarding the negative control. On the other hand, the specimens treated with NaF product showed similar spectra as the positive control.

Table 2 shows the compositional parameters related to the main inorganic/organic components, associated to the 1030/1640 absorption area ( *i.e.,* PO_4_/amide I ratio) and the CI. We observed a significant decrease in the PO_4_/amide I ratio for positive control (Stage 2). On the other hand, after the application of the remineralization products + pH-c (Stage 3), we also observed a significant increase in the PO_4_/amide I, which is relatively lower for the RivaStar product. Moreover, CI increased in all groups treated with remineralizing agents (Stage 3), regarding the negative control group (Stage 1). However, Saforide and Cariestop also significantly increased values regarding the positive control group (Stage 2), while the other products (RivaStar and NaF) showed values similar to the ones of Stage 2.

**Table 2:** Mean (SD) of the inorganic/organic compositional ratios obtained by ATR-FTIR analyses for each experimental stage (n=24; p<0.05; Wilcoxon test)

Experimental Groups	Negative control (Stage 1) 1030/1640	Positive Control (Stage 2) 1030/1640	Treated Dentin+pHc (Stage 3) 1030/1640
Saforide	0.69 (0.19)^a^	0.41 (0.14)^b^	1.43 (0.48)^c1^
Cariestop	0.68 (0.23)^a^	0.45 (0.17)^b^	1.74 (0,63)^c1^
RivaStar	0.63(0.24)^a^	0.36(0.16)^b^	1.18 (0.46)^c2^
NaF	0.58 (0.17)^a^	0.39 (0.15)^b^	1.64 (0.44)^c1^

	**(Stage 1)**	**(Stage 2)**	**(Stage 3)**
	**CI**	**CI**	**CI**

Saforide	0.95 (0.21)^a1,2^	1.17 (0.20)^b^	1.66 (0.76)^c1^
Cariestop	1.06 (0.15)^a1^	1.17 (0.15)^a^	1.42 (0.16)^b1^
RivaStar	0.94 (0.26)^a2^	1.64 (1.02)^b^	1.48 (0.25)^b1,2^
NaF	1.12 (0.17)^a3^	1.26 (0.13)^b^	1.40 (0.37)^b2^

*Different letters indicate significant differences in rows for each parameter.

*Different numbers indicate significant differences in columns for each parameter

Negative control (Stage 1) → Sound dentin; Positive control (Stage 2) → Demineralized dentin; treated dentin + pH-cycling (pH-c) (Stage 3) → SDF/NaF treated dentin under acid challenge. CI (Crystallinity Index)

### X-ray Diffraction (XRD)

**Figure 3:**
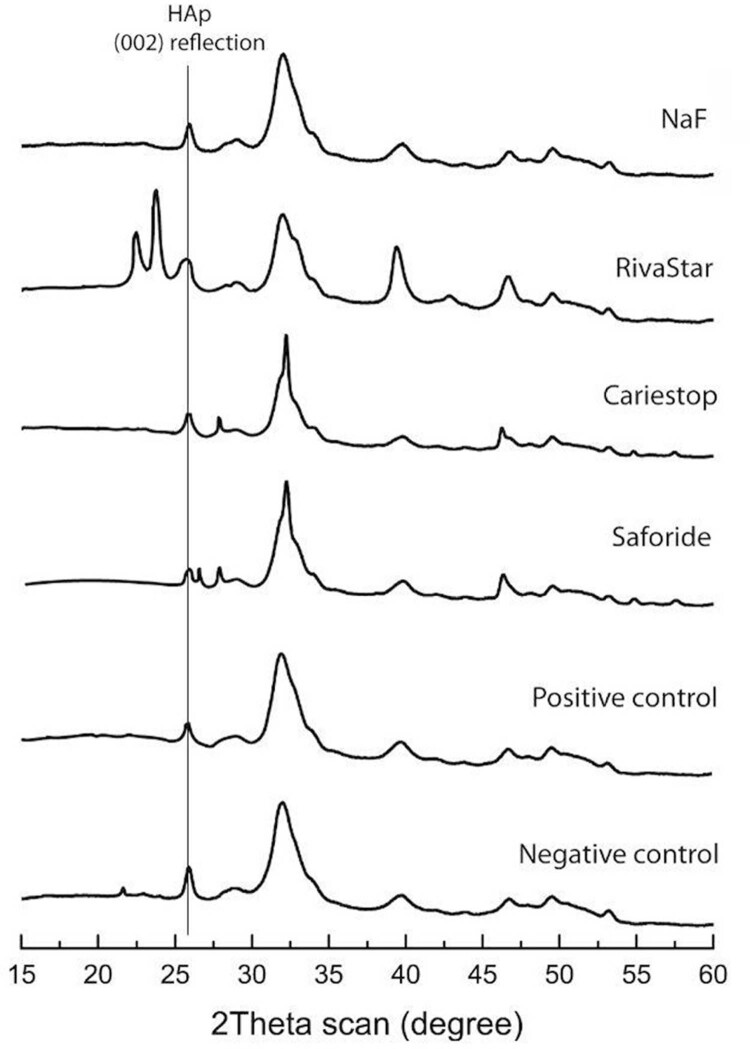
X-Ray diffraction patterns (2Theta scan) for negative and positive controls and SDF/NaF experimental groups. The HAp crystallite size was determined by measuring the FWHM values for the (002) reflection (i.e., corresponding to the c-axes)

We studied the identification of the crystalline phases precipitated during remineralizing treatments and the crystallinity properties of the dentin mineral by means of XRD analyses. Figure 3 shows the integrated 2Theta patterns (one-dimensional scanning) for each of the treatments and the control groups. The diffraction patterns show highly crystalline phases formed due to the remineralizing treatments on the dentin surfaces, corresponding to silver chloride (chlorargyrite, AgCl) for Cariestop and Saforide agents and silver iodide (iodoargyrite) for RivaStar and NaF crystalline phase. Table 3 shows crystallite size measurements for both controls (Stage 1 and Stage 2) and experimental groups (Stage 3). We determined the crystallite size of the dentin mineral by measuring the full width at half maximum (FWHM) for the 002 diffraction line ( *i.e.,* corresponding to the c-axes) for hydroxyapatite (HAp) crystals. These results indicate that the crystallite size of HAp crystals in the negative control (Stage 1) decreased (associated to a higher peak widening) compared to positive control (Stage 2). After the application of the remineralization products (Stage 3), we observed an increase in the crystallite size of HAp crystals to similar values corresponding to negative control in all experimental groups, being this increment greater for Saforide product.

**Table 3:** Full width at half maximum (FWHM) and crystallite size (d) hydroxyapatite (HAp) crystals for 002 reflection (c-axes direction) for negative and positive controls and SDF/NaF remineralizing product treatments

	FWHM	d (nm)
Experimental Groups	HAp-002	
Negative control (Stage 1)	0.44	21
Positive control (Stage 2)	0.71	12.6
SDF/NaF products (Stage 3)		
Saforide	0.36	26.33
Cariestop	0.41	23.32
RivaStar	0.59	20.61
NaF	0.45	20.64

### SEM-EDX analyses

**Figure 4:**
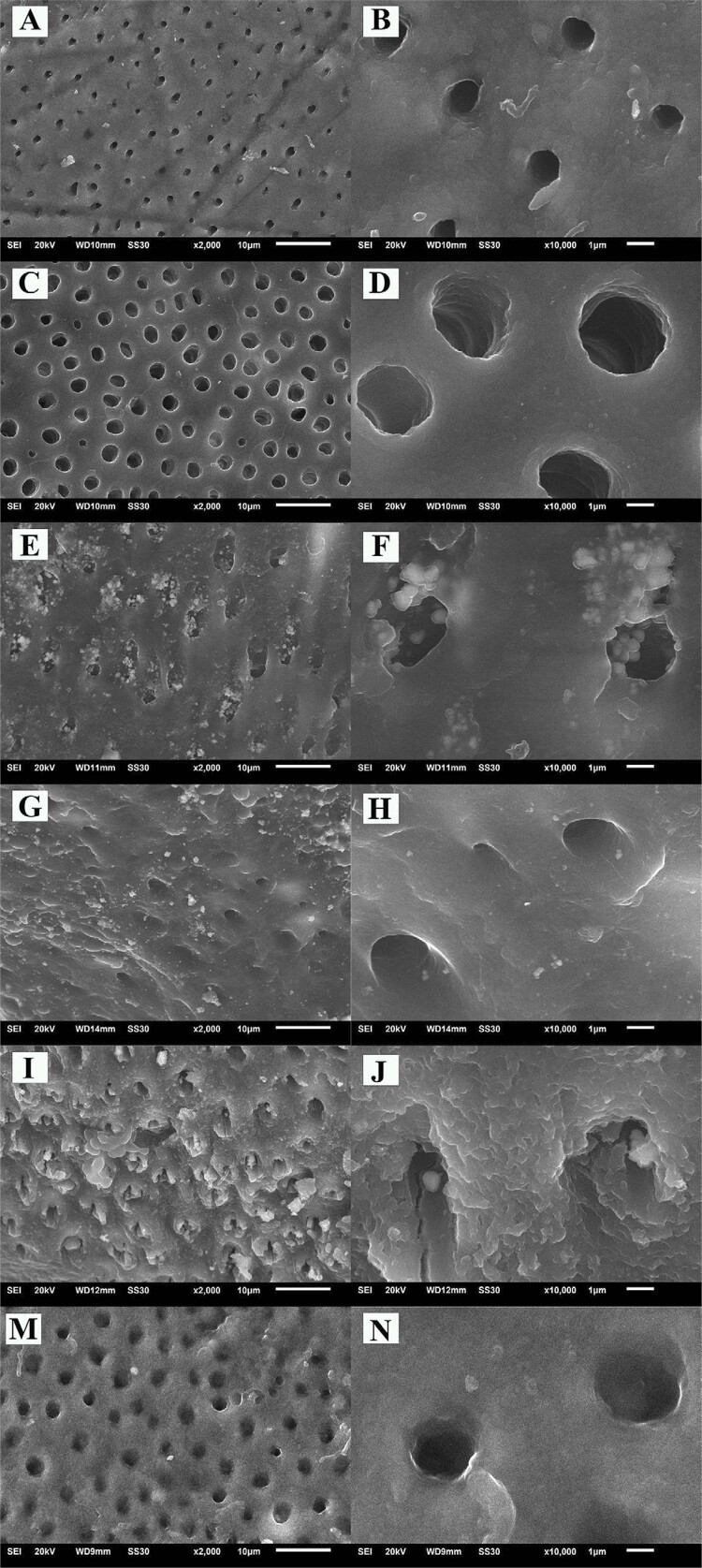
Representative SEM images of sound dentin negative control (A-B) and demineralized dentin positive control (C-D). Experimental groups after the application of remineralizing products Stage 3: Saforide (E-F), Cariestop (G-H), RivaStar (I-J), and NaF (M-N)

Figure 4 shows representative SEM images at different magnification (10.000x–30.000x) for each experimental stage. The images for negative control (Stage 1) ( Figure 4A-B ) show the characteristic of morphological appearance of dental tubules, with a smooth and regular surface. On the other hand, the positive control (Stage 2) ( Figure 4C-D ) shows a rougher surface with an extensive opening of some dentinal tubules. Dentin surface treated with the different SDF/NaF remineralizing agents (Stage 3) ( Figure 4E-N ) showed crystalline precipitates (previously described in the XRD analysis) covering the surface and partially filling the dentin tubules. Between them, the RivaStar experimental group ( Figure 4I-J ) presented a greater precipitation number in the intertubular dentin and peritubular dentin compared to the other SDF experimental groups. Dentin surface treated with NaF showed no apparent precipitation ( Figure 4M-N ).

**Figure 5:**
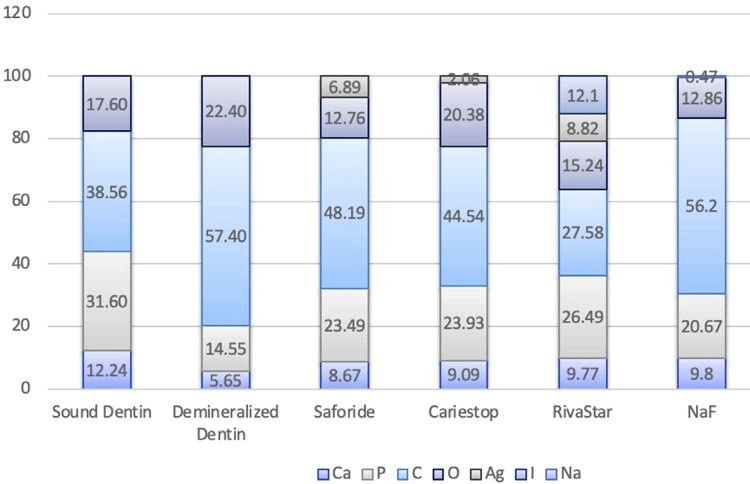
Elemental distribution of P, Ca, C, O, Ag, I, and Na in the dentin surface by EDX semi-quantitative point analysis for negative and positive controls and SDF/NaF remineralizing product treatments

Figure 5 shows the results of the chemical analysis obtained by EDX. These semiquantitative analytical data showed the relative loss of the elements associated with the HAp mineral component (i.e., Ca and P percentages) in the positive control (Stage 2), although the Ca/P ratio was like that observed for negative control (Stage 1) (approx. ~ 2.57). The relative concentrations of these elements increased in the groups treated with remineralizing products (Stage 3) (i.e., Ca>23.50% and P>8.67%, respectively), showing higher Ca/P ratios for the SDF treatment groups (above 2.63). Furthermore, all groups treated with SDF products also presented silver (Ag), although the products’ distribution was heterogeneous on the dentin surfaces. Finally, iodine (I) was detected in RivaStar, as well as Na in the NaF treatment group.

### Three-point bending test

Table 4 shows the mean values of flexural strength for each experimental stage. The specimens treated with the different SDF/NaF products (Stage 3) increased flexural strength compared to the control groups (Stage 1 and Stage 2). Regarding the mechanical response between the remineralizing products, Saforide had the highest flexural strength values, although not significantly different from Cariestop and NaF. On the other hand, RivaStar presented the lowest values, also not statistically significantly different from Cariestop and NaF.

**Table 4:** Flexural strength values (mean ± S.D.) determined by three-point bending test for negative and positive controls and SDF/NaF remineralizing product treatments

Experimental Groups	Mpa
Negative control (Stage 1)	50.71(3.40)^a^
Positive control (Stage 2)	47.30(3.53)^a^
SDF/NaF products (Stage 3)	
Saforide	63.17(4.36)^bcd^
Cariestop	55.86(3.32)^ac^
RivaStar	49.18(2.87)^a^
NaF	55.26(3.90)^ad^

*Different letters indicate significant differences between the groups. (n=20; Kruskal–Wallis and Mann Whitney U tests; p<0.05)

## Discussion

The present study investigated the remineralizing capacity of commercial SDF/NaF products on demineralized dentin exposed to a cariogenic challenge. Our results indicate that the application of these products increased the mineral/organic content and the crystallite size of the hydroxyapatite crystals in the SDF/NaF treated dentin. We associated these compositional and microstructural alterations, as well as the formation of different crystalline phases on the dentin surfaces, with a partial improvement in the mechanical properties of the remineralized dentin. These products promote many effects in the dentin properties that resisted an experimental acid challenge (induced by pH-c) and, thus, they preserve the remineralizing capacity of the SDF/NaF and prevent demineralization of the dentin structure. In this way, we rejected the two hypotheses since the results showed differences between control (sound and demineralized dentin) and SDF/NaF treatment groups considering the different experimental stages.

One of the main limitations in the application of *in vitro* models in Cariology is their limited capability to reproduce some interindividual factors of the intraoral environment (e.g., plaque pH values, saliva, and microbial composition). However, these models have important advantages regarding the high level of scientific control and the consequent lower intrinsic variability and, thus, the smaller sample size required. *In vitro* simulation of dental caries using a standardized model, such as the pH-c, is an accepted method in dental research due to its ease of technique and reproducibility.^26 , 28 , 29^ The pH-cycling method is widely used for many studies on dentin caries, with slight modifications depending on the study objectives.^25 , 26^ For example, pH-c models were employed to detect the dose and pH responses of fluoride dentifrices and their association with other anticaries treatments.^30^ Previous studies have also described that pH-c models can simulate mineral alterations at the compositional, microstructural and morphological levels, which resemble the characteristics of natural caries.^25^ In our study, samples were treated with different remineralizing products in previous demineralized dentin and subjected to a subsequent pH-c protocol to evaluate their potential remineralization capacity under acidic conditions (simulating caries formation). Nevertheless, this experimental study did not consider all possible oral factors; in particular, the complexity of any tooth-plaque-saliva interface was not simulated^31 , 32^, which may present some limitations for extrapolation to clinical situations.

Understanding the characteristics of the mineralized structures in dental tissues is essential to evaluate possible treatments for clinical application and their potential response *in vivo* . Transversal microradiography (TMR) is the gold standard technique to evaluate in depth demineralization of dentin and enamel samples.^32 , 34^ However, the methodological procedure for sample preparation (e.g., sawing/polishing, particularly challenging for brittle samples) complicates the application of this technique in experimental designs that require a sequential analysis of the same specimens, as in our study. Furthermore, in the present study, possible artifacts due to radiodensity/radiopacity associated with the presence of other crystalline phases in SDF treatments (such as chlorargyrite and iodargyrite)^35^ may misinterpret the remineralization effects associated with the HAp phase precipitation in the mineral dentin. This study aims to use complementary non-destructive techniques, such as ATR-FTIR and XRD, to analyze the physicochemical properties of the remineralized dentin surfaces.

Several conditions may work for remineralization of demineralized dentin: (1) presence of residual mineral precipitates acting as crystal growth centers, (2) sources containing calcium and phosphorus for potential mineral precipitation, and (3) maintenance of collagen structure as a scaffold for controlled crystal growth (33). The ATR-FTIR results revealed how remineralization treatments based on SDF/NaF products influenced the chemical characteristics of the demineralized dentin. The infrared spectra obtained for each of these treatments showed relative absorption variations of molecular groups associated with bands related to various organic and inorganic components of the mineral dentin ( Figure 1 ). A closer look at the ATR-FTIR spectra ranging from 900 cm^- 1^ to 1200 cm^- 1^ (v_1_,v_3_ PO_3_^- 4^; phosphate group) showed a decrease in the relative absorption and a variation of the band profile shifted to higher wavenumbers, associated to different crystalline environment of the dentin mineral as a response of the demineralization and remineralization processes.^36^ On the other hand, the effect of ammonia solutions during the application of SDF products decreased relative intensity related to ~1633 cm^- 1^ (amide I band, most abundant fibrous-collagen protein), probably due to an increase of the covalent bonds within the collagen structure.^37^ However, this phenomenon is absent in the RivaStar group, probably due to the KI addition in their composition and the absence of ammonia in the NaF product. Similar effects were also observed for 1544 cm^- 1^ (amide II – peptide C-N stretching and NH bending vibrations) and 1235 cm^- 1^ (amide III bands – CN stretching and NH bending, CC stretching and CH bending vibrations).^38 , 39^ These different changes in the amides groups observed in SDF products suggest a possible alteration in the organization of the fibrous collagen structure of the macromolecular organic network and a dehydration effect of the collagen fibers.^40 , 41^ Thus, SDF treatment generates a collagen cross-linking effect that influences the mineral precipitation mechanisms of these products and, ultimately, affects the mechanical properties of the dentin structure. Moreover, the inorganic/organic components (expressed as 1030/1640 ratio) indicated that after the application of the SDF/NaF products (Stage 3) the phosphate content relatively increased, even under pH-cycling conditions (i.e., caries simulation). The SDF reacts with hydroxyapatite composing mineral dentin to form calcium fluoride and insoluble silver phosphate salts, increasing the resistance to acid dissolution.^13 , 20^ The remineralization capacity of the SDF can be attributed to its alkaline pH, as well as the large amount of the fluoride content (44,800 ppm), resulting in the formation of fluorapatite, which can act in a synergistic process to promote remineralization.^42^ Furthermore, we detected an alteration in the CI of the hydroxyapatite mineral obtained by ATR-FTIR, probably due to the selective dissolution/precipitation of phosphate groups (lower and higher crystallinity vibrational environment), during the different experimental stages. In the Stage 2 of pH-c, the CI increased compared to the negative control (Stage 1), as a result of the preferential dissolution of lower crystallinity phosphate components, due to the caries process simulation.^33^ On the other hand, after the application of the remineralizing products, the phosphate environments of the mineral dentin selectively increased in higher crystallinity phosphate compounds and a possible greater dissolution of lower crystallinity phosphate occurred by the subsequent pH-c (Stage 3). These compositional variations may influence the microstructural characteristics of the hydroxyapatite crystals in dentin during the different experimental stages.

The crystalline properties during the remineralization process in the application of SDF/NaF products may determine the morphological and mechanical properties of the dentin. Previous studies show that pH-c can simulate the mineral characteristics of the natural carious dentin process,^25^ as also observed in the positive control (Stage 2) of the present study. Our earlier *in vitro* study demonstrated that the immediate application of SDF products resulted in the formation of different crystalline phases of silver salts (i.e., AgCl and AgI compounds).^20^ On the other hand, we obtained the crystallite size of the dentin mineral from the (002) diffraction line in the XRD pattern, corresponding to the c-axes direction of the HAp crystals. Other reflections were also detected in the SDF groups associated with the crystalline phases of chlorargyrite (Saforide and Cariestop) and iodargyrite (RivaStar), suggesting that these compounds precipitated as a separate phase in the SDF-treated groups.^20 , 43^ Moreover, previous studies showed that SDF compounds react with calcium and phosphate ions to produce fluorohydroxyapatite crystals.^44^ The alkaline conditions produced by SDF treatments are also favorable for synthesizing fluorohydroxyapatite, which can accelerate the reaction process by promoting precipitation.^45^ Furthermore, the apatite crystal sizes and/or the crystal lattice perfection accompany fluoride uptake in physiological solution.^46^ We observed crystallite sizes in the SDF/NaF products, particularly in Saforide, related to the specific c-axis of apatite crystals. These anisotropic properties in the crystalline development of apatite may also influence the chemical stability of the precipitates formed during SDF/NaF treatments. After the application of the mineralizing agents, pH-c shows how the crystalline characteristics of the precipitates remain roughly unchanged compared to the SDF immediate application.^8^ However, the compositional characteristics of these calcium phosphate phases (i.e., crystalline hydroxyapatite) are associated with higher crystallinity phosphates environments (CI, obtained by ATR-FTIR analyses previously described). In general, these physico-chemical mechanisms during the remineralization process benefits different factors, such as mineral solubility and crystalline characteristics, which ultimately establish its biomechanical properties.

In the SEM images, we observed the regular morphology with a rough surface in the sound dentin (Stage 1) ( Figure 4A-B ).^47^ After pH-c, we detected a higher aperture of dentinal tubules and a smoother surface, which coincided with the characteristics of demineralized dentin (Stage 2) ( Figure 4C-D ).^24^ The application of the SDF products (i.e., Saforide, Cariestop, and RivaStar) resulted in the formation of different crystalline phases, heterogeneously distributed on the dentin surface and partially occluding the dentinal tubules ( Figure 4E-N ). Particularly in RivaStar group, the precipitation of silver particles increased occlusion of the dentin tubules. These observations have been previously described in SDF treatment applications associated with areas of greatest dentin demineralization.^13^ The EDX analysis ( Figure 5 ) corroborates that these precipitations are silver particles, corresponding to crystalline phases of chlorargyrite (Saforide and Cariestop) and iodargyrite (RivaStar), as previously observed using XRD analyses. On the other hand, dentin treated with NaF showed a rough surface ( Figure 4.M-N ), in which we did not find the formation of any crystal precipitation, as has been previously observed using similar treatments.^48 , 49^ Moreover, an increase in the Ca/P ratio after the application of SDF products suggested that occurred remineralization of the previously demineralized dentin. Similar remineralization processes using SDF applications have been reported,^23 , 50^ SDF/KI fluoride concentrations inhibited demineralization and/or enhanced remineralization of artificial dentin lesions. These overall alterations in the morphological, compositional, and structural properties of the dentin due to remineralizing products can lead to changes in its mechanical response.

One criterion for assessing dental tissue remineralization is the evaluation of its mechanical properties.^45^ The demineralization in a carious process reduces the mineral/organic content of the dental tissues and, consequently, the mechanical properties of the tooth may be impaired.^26 , 51^The inorganic phase provides strength, whereas the organic phase is responsible for the toughness of dentin structure.^26^ Our results demonstrated that the experimental groups treated with SDF/NaF products (Stage 3) improved the mechanical properties of the demineralized dentin, even after the caries simulation by pH-c. Furthermore, a higher concentration of SDF generated a greater inhibitory effect on MMPs,^17^ which may contribute to avoid collagen degradation, reinforcing the mechanical properties. Moreover, the improvement in the mechanical properties could be also attributed to the alteration in the mineral/organic content and its crystalline characteristics, confirmed with ATR-FTIR and XRD analyses. On the other hand, RivaStar group showed the lowest increase in the flexural strength values, suggesting that the combination of SDF and potassium iodide (KI) may affect properties of SDF. In fact, the presence of KI decreases the amount of silver compounds on the dentin surface, which negatively affects its mechanical response.^13 , 50^These mechanical properties may critically influence the success of the future restoration using these remineralizing products.

The application of SDF products is a simple, non-invasive, and affordable treatment for dental caries. Moreover, SDF is also a potential agent in the non-restorative control of dental caries.^14 , 52^ The present study reveals how the alteration of the physicochemical properties of dentin treated with SDF/NaF improves the mechanical properties of the dentin surfaces under pH-cycling conditions, simulating a dental caries process. Thus, the remineralization capacity of these treatments, due to the potential reaction of their chemical compounds with the hydroxyapatite of the dentin mineral,^8^ appeared to show a mechanical effect on demineralized dentin that favors the stability of the treatment under acidic conditions (i.e., cariogenic challenge). Based on these results, we observed that the Saforide reacted better than Cariestop and RivaStar, both presenting a similar fluoride high concentration between the SDF products. On the other hand, we used the NaF product at a very low fluoride concentration compared to the other fluoride sources. Therefore, the effect of 0.2% sodium fluoride solution (~850 ppm fluoride) should be directly contrasted with the SDF products (~40.000 ppm fluoride). In the present study, even at a very low fluoride concentration, this product significantly changed the hydroxyapatite crystalline structure. These findings are corroborated by other studies, which concluded that the use of 0.2% NaF can decrease caries.^9 , 10^These differences provide important information for clinicians on the use of the appropriate material for each patient. However, this experimental study was insufficient to determine the impact of the *in v* ivo antimicrobial activity attributed to these products. In future studies, it would be interesting to determine the long-term evaluation of these products in caries processes (i.e., progression under pH cycling conditions), as well as the aesthetic issue related to the application of these products.

## Conclusions

The use of silver diamine fluoride and 0.2% sodium fluoride solution has a remineralizing effect on the dentin surface even under acid attack. Despite the fluoride concentration, the application of these products causes the precipitation of different crystalline salts, which is associated with an increase in the mineral/organic content and an alteration of the crystalline characteristics of dentin, thus, ultimately increasing the mechanical properties of demineralized dentin. Within the silver diamine fluoride products, the crystalline properties (higher CI and larger crystallite size) in Saforide treatment seem to be related to a higher mechanical response after acid attack. Due to the limitations of this study, further research in settings containing all oral factors to evaluate its clinical application is essential.
